# Barriers to COVID-19 vaccine uptake: classification and the role of Health Literacy and Media Literacy

**DOI:** 10.3389/fpubh.2023.1238738

**Published:** 2023-11-08

**Authors:** Soheila Ranjbaran, Khalil Maleki Chollou, Sara Pourrazavi, Towhid Babazadeh

**Affiliations:** ^1^Department of Public Health, Sarab Faculty of Medical Sciences, Sarab, Iran; ^2^Department of Nursing, Sarab Faculty of Medical Sciences, Sarab, Iran; ^3^Research Center of Psychiatry and Behavioral Sciences, Tabriz University of Medical Sciences, Tabriz, Iran

**Keywords:** barriers, COVID-19, vaccine, Health Literacy, Media Literacy, Iran

## Abstract

**Background:**

Vaccination is one of the most influential and cost-effective health interventions for preventing and reducing COVID-19 diseases. Unfortunately, the majority of the world's population is deprived of vaccination. Health Literacy (HL) and Media Literacy (ML) are essential to the COVID-19 vaccination. The present study investigates the barriers to COVID-19 vaccine uptake, focusing on classification and the roles of HL and ML.

**Methods:**

A cross-sectional study was conducted among people 18–65 years old in Sarab City, located in East Azerbaijan, Iran, between September to October 2020. Multistage cluster sampling was employed to recruit 298 people from Health Care Services Centers (HCCs).

**Results:**

The results of this research demonstrated that about 32.6% of participants reported that they have fully injected COVID-19 vaccines. Also, HL was positively associated with ML (*r* = 0.214, *p* < 0.05). Barriers of COVID-19 vaccine uptake were classified into personal, interpersonal, group and organizational, society and decision-making factors. Besides, barriers to the COVID-19 vaccine were significantly correlated with HL (*r* = −0.298, *p* < *0*.05) and ML (*r* = 0.266, *p* < *0*.05). Additionally, in the hierarchical regression model, demographic characteristics accounted for 8.2% of the variation in barriers to the COVID-19 vaccine (*F* = 4.34; *p* = 0.001), that monthly income (ß = −0.237; *p* < 0.05) and marriage statues (ß = 0.131; *p* < 0.05) were statistically associated with low barriers. HL as predictor variables explained an additional 14.4% of variation in barriers of COVID-19 vaccine (*F* = 53.84; *p* < 0.001) and ML explained an extra 9.2% of the variation (*F* = 38.83; *p* < 0.001). In total, demographic characteristics, HL dimensions and ML were able to explain 31.8% of the variation in barriers to COVID-19 vaccine.

**Conclusions:**

According to the findings, various strategies are needed to increase the COVID-19 vaccination uptake. This is due to the fact that barriers to COVID-19 vaccination uptake are multifactorial. These facts can help health policymakers and healthcare providers design media-based interventions to reduce barriers to COVID-19 vaccination uptake among adults. Enhancing vaccine HL and ML for adults and improving vaccine confidence are of high priority.

## 1. Introduction

The COVID-19 pandemic has led to significant changes in people's daily lives worldwide (1). Since the COVID-19 outbreak, many precautions have been implemented, including quarantines, lockdowns, wearing mandatory face masks in public, and social isolation (2, 3). Vaccination is one of the most crucial and cost-effective health interventions (4) for achieving herd immunity and halting the COVID-19 pandemic (1). At least 80% of the population must be vaccinated for herd immunity (5). However, there are some perceived barriers to achieving the desired vaccination coverage. According to a systematic review, worldwide acceptance rates for the COVID-19 vaccine ranged from 97% to 23.6%, with the lowest rate reported in the Middle East (6).

According to the health belief model, perceived barriers can directly lead to non-participation in preventive behavior (5). The literature has shown that insufficient information about vaccines and their side effects, mistrust in the source of the vaccine (6), concern and mistrust about the vaccine's efficacy and safety, and belief in natural immunity (7) are perceived barriers to COVID-19 vaccination. Studies with similar results have also been conducted in Iran (8–10). In this regard, Jahanshahi-Amjazi et al. found that 58% of Iranians faced barriers to receiving the COVID-19 vaccine (8). Khankeh et al. (9) identified insufficient trust in the healthcare system, vaccine safety, and adverse effects as primary barriers to vaccine acceptance. Many perceived barriers to COVID-19 vaccination can be influenced by insufficient knowledge and misinformation about the vaccine and poor HL (11).

Misinformation has spread as quickly as the development of the vaccine, primarily through social media (12). Misinformation spreads faster than correct information, and more audiences are exposed to it (13). The World Health Organization (WHO), in response to the rapid expansion and high volume of false information published via digital social media, has coined the term “infodemic” to describe “too much information, including false or misleading information, in digital and physical environments during a disease outbreak” (14). False and misleading information about the COVID-19 vaccine can be challenging for people with insufficient HL and ML and cause them to hesitate or refuse vaccination (11). That is why WHO has requested all countries to fight the infodemic (15).

HL is “the degree to which individuals can obtain, process, understand, and communicate health-related information needed to make informed health decisions” (16). Various personal, demographic, social and informational factors such as gender, language, ethnicity, health education, physical condition, marital status, place of residence, and access to information sources can influence HL level (14, 17, 18). Inadequate HL is associated with adverse health outcomes and less use of preventive services (11). It is considered one of the essential psychosocial factors in the formation of health behaviors (19). According to studies, people with low HL have less intention to receive the COVID-19 vaccine or are hesitant about it (11, 20).

Due to the critical conditions caused by the COVID-19 pandemic and the urgent need for mass vaccination, COVID-19 vaccines were produced and distributed at an unprecedented speed (11). The rapid production and distribution of vaccines caused various information sources, such as social media, to publish information about the inefficacy and non-safety of vaccines (21). Much of this information may need to be revised (21). Therefore, if people cannot evaluate and understand this information, they will be confused, hesitant, and refuse to get the vaccine (12). Recent studies showed that social media play a role in public vaccination refusal (22), indicating the necessity to enhance public ML to analyze media contents related to COVID-19 vaccination (20). The WHO considered ML one of the top five literacy skills (12). ML means thinking about and retrieving correct information and determining its accuracy and correctness for personal or collective use (12). Given the proliferation of anti-vaccination conspiracy theories through social media, ML is undoubtedly a vital tool for public health (23).

According to the background mentioned, studying HL and ML as factors affecting barriers to COVID-19 vaccine uptake can be important for several reasons. By studying HL and ML, we can understand how people access and process information about vaccines, which can influence their decision-making process to uptake (12). As such, considering the rapid dissemination of misinformation and vaccine-related myths through diverse media platforms, comprehending Media Literacy (ML) has a crucial role. By understanding ML, we can effectively identify and combat misinformation, empowering individuals to make informed choices based on credible evidence regarding the COVID-19 vaccination (12). Identifying Health Literacy (HL) and Media Literacy (ML) as factors influencing barriers to COVID-19 vaccine uptake holds significant potential in developing targeted interventions, enhancing communication strategies, and combatting misinformation. These endeavors are crucial for fostering vaccine acceptance and safeguarding public health. The results of this research can effectively gain valuable insights that can guide effective measures in promoting widespread vaccination. Hence, this study aimed to investigate barriers to COVID-19 vaccine uptake by examining the classification and roles of HL and ML.

## 2. Methods

### 2.1. Study design and participants

We conducted a cross-sectional study on 298 participants aged 18–65 in Sarab, East Azerbaijan, Iran, between February to May 2023. The sample size was calculated based on information from a similar study (24) and a confidence level of %95, *Z* = 1.96, SD = 2.89, Mean = 6.74, 283 samples. Finally, considering the possibility of dropping samples, 300 people were included in the study, of which 293 completed the questionnaires completely. Multistage cluster sampling was employed to recruit the participants from Healthcare Services Centers (HSCs). The city of Sarab includes four health centers, each of which was considered a cluster. Then, in the first sampling stage, each HSCs were considered a cluster. In the second stage, participants were randomly selected from the four HSCs (75 people from each HSCs) according to their health records. Respondents were invited to participate in the survey by phone, informed about the research objectives, and signed a formal informed consent form. Participants answered the questionnaire items in a consultation room at the health center. Because of the nature of the study questions and the culture of the study population, all interviews were performed by two trained interviewers to make participants feel at ease. Individuals aged 18–65 with consent to participate in the study were required for inclusion. Exclusion criteria amounted to failing to complete the questionnaire completely and correctly.

### 2.2.Measure

Published instruments were applied to collect the following data. A brief description of the questionnaire is as follows:

#### 2.2.1. Demographic information questionnaire

Demographic information includes participants' gender, age, marital status, job status, education level, and income status.

#### 2.2.2. Health literacy for iranian adults (HELIA)

We used the validated Health Literacy for Iranian Adults (HELIA) (25). This questionnaire consists of 47 items and five dimensions: (1) Reading health information (4 items) is measured using a five-interval Likert scale, ranging from 1 (completely difficult) to 5 (completely easy). The total score ranged from 4 to 20. The higher scores represented a high level of reading health information. (2) Understanding health information (7 items) was rated on a 5-point scale ranging from 1 (completely difficult) to 5 (completely easy). The scores for understanding items ranged from 7 to 35. The higher scores determined the better condition for understanding; (3) Appraisal of health information (4 items), rated on a 5-point scale ranging from 1 (never) to 5 (always). The total scores for this dimension ranged from 4 to 20. The high level of scores indicated an increased ability to appraise health information; (4) Ability to access health information (6 items) was scored based on a five-interval Likert scale (always = 5, most of the time = 4, sometimes = 3, seldom = 2, and never = 1). The scores ranged between 6 and 30, and a higher score indicated a better ability to access health information; (5) Decision making (12 items) was measured on a five-interval Likert scale (always = 5, most of the time = 4, sometimes = 3, seldom = 2 and never = 1). The scores for decision-making items ranged from 12 to 60; the higher scores indicated a better condition. Cronbach's alpha for all of the dimensions of the questionnaire was >0.7 (0.72–0.89).

#### 2.2.3. Media literacy scale

A validated and reliable scale was used to assess participants' ML (26). The Cronbach's α of this scale was between 0.75 and 0.8. The questionnaire consists of 20 items with five subscales: “understanding the contents of a media message”, “realizing the hidden objectives of a media message”, “deliberate choices of media messages”, “critical view on media messages”, and “analysis of media messages”. The items were rated using a five-point Likert scale (1 = strongly disagree, 2 = slightly disagree, 3 = not sure, 4 = slightly agree, 5 = strongly agree). The overall score ranged between 20 and 100.

#### 2.2.4. Barriers of COVID-19 vaccine

A researcher-made questionnaire assessed barriers to COVID-19 Vaccine uptake. The questionnaire was prepared by reviewing other questionnaires applied in similar studies (7, 24, 27–30). The validity of the questionnaires was assessed by an expert panel (four health educationists, a socialist, and an epidemiologist). To assess reliability, a pilot study was performed on 25 people not included in the final sample. Finally, the version of the scale included 33 items with four dimensions as follows: intrapersonal factors (15 items; α = 0.87), Interpersonal factors (3 items; α = 0.77), Group and organization factors (5 items; α = 0.79), and Society and Policy-making factors (10 items; α = 0.76). For all dimensions, the items were rated on a 5-point Likert-type scale ranging from 1 to 5 (5 = totally agree through 1 = totally disagree). The total score ranges from 33 to 165; the higher score indicates more barriers to COVID-19 vaccine uptake by the participants. The Cronbach's α for all questionnaire, was measured 0.92.

#### 2.2.5. COVID-19 vaccine uptake

Two questions were used to evaluate participants' performance in taking the COVID-19 vaccine [i.e., did you get the COVID-19 vaccine? (Yes or No)]; How many doses of the COVID-19 vaccine did you take? (1 dose; two doses and more).

### 2.3. Data analysis

We conducted all analyses using SPSS 21 (SPSS Inc, Chicago, IL, USA). The data were presented as mean (SD) for quantitative variables and frequency (percent) for qualitative variables. To assess normality, we utilized the Kolmogorov-Smirnov test. We employed the independent sample *t*-test and one-way ANOVA for bivariate comparisons of quantitative variables. Additionally, the relationship between HL and ML with barriers to the COVID-19 vaccine was measured using Pearson correlation.

In three steps, we applied Hierarchical Linear Regression analysis for barriers to the COVID-19 vaccine. The demographic variables, including age, gender, education level, job, income and marriage, were entered in the first step. HL was involved in the second step, along with the demographic variables, and in the third step, we entered the demographic variables and HL with ML in the analysis. To determine the percentage of variance characterized by barriers, we assessed the adjusted R2 change following the insertion of each block. The threshold for significance was fixed at = 0.05. The significance level was set to α = 0.05.

## 3. Results

The demographics of the participants are presented in Table 1. Among the 298 participants, 41.6 % were in the age group of 30 to 39 years. Most participants were male (55%), with an education level of diploma or under (51.7%). More than half of the participants were undergraduates (6.88% freshmen, 46.50% sophomores, 25.13% juniors, and 9.93% seniors). Over 37% of participants reported a monthly family income between 150 and 200 dollars or more. About 32.6% of participants reported that they had been fully injected with COVID-19 vaccines.

**Table 1:** Relationship between HL, ML, and barriers to the COVID-19 vaccine and some of the demographic characteristics.

**Variables**		***N* (%)**	**HL**	***P*-value**	**ML**	***P*-value**	**Barriers to COVID-19 vaccine**	***P*-value**
			**Me** ±**SD**		**Me** ±**SD**		**Me** ±**SD**	
Age (year)^*^	18–29	105 (35.2)	71.01 ± 18.51	0.259	59.94 ± 17.12	0.945	113.22 ± 24.67	0.332
	30–39	124 (41.6)	71.83 ± 19.52		60.45 ± 15.28		109.22 ± 24.24	
	40 and higher	69 (23.2)	75.86 ± 22.21		60.71 ± 19.67		108.14 ± 25.85	
Gender^**^	Male	164 (55)	75.35 ± 18.13	0.007^***^	60.53 ± 15.12	0.837	109.46 ± 22.48	0.413
	Female	133 (45)	69.01 ± 21.41		60.12 ± 19.11		111.83 ± 27.28	
Education level^*^	Diploma and under diploma	154 (51.7)	67.72 ± 19.37	0.001^***^	59.44 ± 15.08	0.384	112.96 ± 23.84	0.084
	Bachelor and higher	144 (48.3)	76.92 ± 19.33		61.16 ± 18.58		108.01 ± 25/47	
Occupation^**^	Unemployed	83 (27.9)	67.46 ± 19.96	0.025^***^	59.62 ± 13/61	0.502	116.65 ± 21.17	0.015^***^
	Self-employment	86 (28.9)	75.50 ± 18.95		62.68 ± 16.30		105.45 ± 26.20	
	Housewife	48 (16.1)	76.54 ± 20.82		59.10 ± 23.32		113.41 ± 28.54	
	Employed	81 (27.2)	72.01 ± 59.23		59.23 ± 16.40		107.44 ± 23.11	
Income (month)^*^	≤ 150 dollars	77 (25.8)	70.88 ± 22.17	0.198	59.49 ± 16.93	0.041	114.91 ± 26.71	0.002^***^
	150–200 dollars	112 (37.6)	71.65 ± 17.16		58.32 ± 15.76		111.49 ± 22.96	
	200 dollars ≤	109 (36.6)	75.94 ± 19.90		64.44 ± 18.21		102.38 ± 22.78	
Marriage^**^	Single	96 (32.2)	67.93 ± 16.97	0.003^***^	59.39 ± 14.71	0.512	115.21 ± 24.71	0.021^***^
	Married	202 (67.8)	74.63 ± 20.79		60.77 ± 17.97		108.12 ± 24.55	

^*^One way ANOVA test.

^**^Independent test.

^***^P < 0.05.

This study established that barriers to COVID-19 vaccination uptake were categorized as personal, interpersonal, group and organizational, societal, and decision-making factors. Questions related to barriers to COVID-19 vaccine uptake are shown in Table 2; of the most important barriers to COVID-19 vaccine uptake by the participants can be pointed to “vaccine side effects” and “belief in better natural immunity than vaccine immunity”.

**Table 2:** The frequency of barriers to COVID-19 vaccine uptake.

**Variables**		**Totally agree**	**Agree**	**No idea**	**disagree**	**Totally disagree**
		***N*** **(%)**	***N*** **(%)**	***N*** **(%)**	***N*** **(%)**	***N*** **(%)**
Personal factors	Vaccine side effects occur in the long term.	79 (26.5)	88 (29.5)	79 (26.5)	41 (13.5)	11 (3.7)
	I refuse vaccination because it causes infertility.	13 (4.4)	19 (6.4)	120 (40.3)	97 (32.6)	49 (16.4)
	I don't need vaccination because my immune system is strong.	18 (6.0)	41 (13.8)	55 (18.5)	132 (44.3)	52 (17.4)
	The risk of vaccination is more than its benefits.	37 (12.4)	44 (14.8)	81 (27.2)	101 (33.9)	35 (11.7)
	Natural immunity is better than vaccination.	74 (24.8)	79 (26.5)	51 (17.1)	70 (23.5)	24 (8.1)
	I've already had COVID-19, so I don't need a vaccine because I'm immune.	17 (5.7)	41 (13.8)	63 (21.10)	117 (39.3)	60 (20.1)
	Vaccine causes side effects.	59 (19.8)	107 (35.9)	74 (24.8)	51 (17.1)	7 (2.3)
	Vaccine is not effective	35 (11.7)	69 (23.2)	81 (27.2)	90 (30.2)	23 (7.7)
	I am afraid of syringes	10 (3.4)	23 (7.7)	25 (8.4)	113 (37.9)	127 (42.6)
	I don't have enough time to get the vaccine	9 (3.0)	18 (6.0)	40 (13.4)	141 (47.3)	90 (30.2)
	I not be motivated to live	17 (5.7)	23 (7.7)	25 (8.4)	90 (30.2)	143 (48.0)
	I belie that corona virus does not exist	13 (4.4)	20 (6.7)	40 (13.4)	101 (33.9)	124 (41.6)
	Because I already got COVID-19, that's why I don't go for vaccination	10 (3.4)	27 (9.1)	53 (17.8)	124 (41.6)	84 (28.2)
	I refuse vaccine because of drug interactions	11 (3.7)	38 (12.8)	67 (22.5)	110 (36.9)	72 (24.2)
	I am waiting to see how the vaccine will work in vaccinated people.	22 (7.4)	53 (17.8)	91 (30.5)	87 (29.2)	45 (29.2)
Interpersonal factors	Why should I vaccinate? I know people who have received two doses of the vaccine and even died.	58 (19.5)	45 (15.1)	75 (25.2)	86 (28.9)	34 (11.4)
	My friends do not vaccinate, I follow them.	4 (1.3)	16 (5.4)	39 (13.1)	137 (46.0)	102 (34.2)
	My parents don't let me get vaccinated.	6 (2.0)	6 (2.0)	34 (11.4)	117 (39.3)	135 (45.3)
Group and organization factors	I refuse to get vaccinated because vaccines are not tested enough	44 (14.8)	54 (18.1)	75 (25.2)	99 (33.2)	26 (8.7)
	There are no long-term research results on the effect of COVID-19 vaccine	48 (16.1)	82 (27.5)	88 (29.5)	63 (21.1)	17 (5.7)
	Vaccination centers are always overpopulated	10 (3.4)	38 (12.8)	46 (15.4)	137 (46.0)	67 (22.5)
	Pharmaceutical organizations intentionally spread the virus to sell their medical supplies.	34 (11.4)	38 (12.8)	111 (37.2)	70 (23.5)	45 (15.1)
	Due to the lack of vaccines from reputable vaccination companies, I do not want to inject vaccines.	43 (14.4)	40 (13.4)	83 (27.9)	88 (29.5)	44 (14.8)
Society and policy-making factors	By vaccinating, you fill the pockets of pharmaceutical companies.	34 (11.4)	50 (16.8)	94 (31.5)	80 (26.8)	40 (13.4)
	I do not vaccinate because none of the vaccines have been approved	30 (10.1)	48 (16.1)	79 (26.5)	105 (35.2)	36 (12.1)
	The vaccine is a conspiracy of the other countries with which they want to change our genes.	19 (6.4)	20 (6.7)	98 (32.9)	99 (33.2)	62 (20.8)
	It seems impossible that vaccine been made so quickly	29 (9.7)	38 (12.8)	110 (36.9)	84 (28.2)	37 (12.4)
	The vaccination is to control the population	23 (7.7)	26 (8.7)	111 (37.2)	92 (30.9)	46 (15.4)
	I won't get any vaccines except Pfizer	20 (6.7)	32 (10.7)	77 (25.8)	113 (37.9)	56 (18.8)
	I read on the internet that vaccines contain microchips.	13 (4.4)	32 (10.7)	107 (35.9)	84 (28.2)	62 (20.8)
	In the media, I saw people demonstrating against the vaccine.	12 (4.0)	48 (16.1)	125 (41.9)	70 (23.5)	43 (14.4)
	They advertise in cyberspace that the vaccine is harmful.	23 (7.7)	60 (20.1)	100 (33.6)	78 (26.2)	37 (12.4)
	I do not trust the health system.	22 (7.4)	21 (7.0)	61 (20.5)	117 (39.3)	77 (25.8)

Table 3 indicates that HL and ML scores were higher among participants with a history of COVID-19 vaccine uptake (*p* < 0.05). Also, individuals with a history of COVID-19 vaccine uptake experienced fewer barriers.

**Table 3:** Comparison of HL and ML variables and barriers to the COVID-19 vaccine between vaccinated and unvaccinated individuals.

**Variables**	**Sub groups**	**Mean (±SD)**	**Mean difference (SD error)**	*p*-value^*^
HL	Yes	74.68 ± 20.02	6.67 (2.42)	0.006
	No	67.91 ± 18.87		
ML	Yes	62.33 ± 56.17	6.16 (2.07)	0.003
	No	56.17 ± 16.04		
Barriers to the COVID-19 vaccine	Yes	102.70 ± 23.11	−23.54 (2.62)	< 0.001
	No	126.24 ± 20.36		

^*^P < 0.05.

Table 4 displays the variables' mean scores and standard deviations and the correlation matrix of all the variables in the mediation model. HL was positively associated with ML (*r* = 0.214, *p* < 0.05). Barriers to the COVID-19 vaccine had a significant correlation with HL (*r* = −0.298, *p* < *0*.05) and ML (*r* = 0.266, *p* < *0*.05).

**Table 4:** Bivariate correlation matrix of the relationship among HL and ML and barriers to COVID-19 vaccine uptake.

**Variables**	**1**	**2**	**3**	**Me ±SD**
1= HL	1			72.47 ±19.86
2= ML	0.214^*^	1		60.33 ± 16.97
3= Barriers to COVID-19 Vaccine	−0.298^*^	−0.266^*^	1	110.39 ± 24.78

^*^Correlation is significant at the 0.05 level (two-tailed).

We used the hierarchical regression model to examine the effects of demographic characteristics, HL, and ML on barriers to the COVID-19 vaccine. In step 1, demographic characteristics accounted for 8.2% of the variation in barriers to the COVID-19 vaccine (*F* = 4.34; *p* = 0.001); that is, approximately 8.6% of the variation in barriers to the COVID-19 vaccine is explained by the demographic variables. Table 4 displays that monthly income (ß = −0.237; *p* < 0.05) and marital status (ß = 0.131; *p* < 0.05) were statistically associated with low barriers. HL as predictor variables (step 2) explained an additional 14.4% of the barriers to the COVID-19 vaccine (*F* = 53.84; *p* < 0.001). In step 3, ML was added, which explained an additional 9.2% of the variation (*F* = 38.83; *p* < 0.001). In total, demographic characteristics, HL dimensions, and ML were able to explain 31.8% of the variation in barriers to the COVID-19 vaccine (Table 5).

**Table 5:** Hierarchical linear regression for prediction of barriers to COVID-19 vaccine uptake through demographic characteristics, HL, and ML.

**Variables**	**ß**	**R2 change**	***F* change**	**SE**	***P*-value**
**Step 1**					
Age	0.070	0.082	4.34	2.09	0.001
Gender	0.115			2.93	
Education level	0.027			2.97	
Job	0.063			1.25	
Income	−0.237^*^			−1.93	
Marriage	0.131^*^			3.37	
**Step 2**					
Age	0.056	0.144	53.84	1.93	0.001
Gender	0.029			2.76	
Education level	0.053			2.78	
Job	0.063			1.15	
Income	0.182^*^			1.79	
Marriage	0.070			3.13	
HL	−0.404^*^			0.069	
**Step 3**					
Age	0.056	0.092	38.83	1.81	0.001
Gender	0.039			2.60	
Education level	0.040			2.62	
Job	0.082			1.09	
Income	0.159^*^			1,068	
Marriage	0.075			2.94	
HL	−0.267^*^			−0.070	
ML	−0.333^*^			−0.078	
Total R2	-	0.318	-	-	-
Adjusted R2	-	0.300	-	-	-

^*^P < 0.05.

## 4. Discussion

The present research investigated barriers to COVID-19 vaccine uptake and the role of HL and ML in public vaccine refusal. About 32.6% of participants reported that they had been fully injected with COVID-19 vaccines. A similar study revealed that 26.5% and 26% of low-concern states and municipalities, respectively, received full vaccinations (23). Another study by Li et al. (31) in China indicated that 41.2% of medical students had vaccine hesitancy. Wirawan et al. found that Indonesia's COVID-19 booster vaccine acceptance rate was 56.3% (32). Various studies have reported the relationship between vaccination and hesitancy. In a systematic review in 2021, the range of COVID-19 vaccine acceptance rates was reported to be between 97% and 23.6% globally, with the lowest rate in the Middle East (33). Due to new strains of the COVID-19 virus, vaccine uptake hesitancy could be a significant problem in efforts to control the COVID-19 pandemic. Hence, identifying the barriers to vaccine uptake and designing media-based interventions can help improve this situation.

There was a relationship between gender, education level, occupation, marital status, and HL, but ML was not associated with demographic characteristics. Previous studies revealed that age, gender, education level, occupational status, income, and marital status had statistically significant associations with e-HL and HL in adults (34, 35). During pandemics, it is essential to improve HL based on socioeconomic status so that people can use accurate information to adopt preventive behaviors. ML may enhance health-related information more publicly than HL; therefore, ML was not associated with socioeconomic status.

The founding of this study demonstrated that barriers to COVID-19 vaccine uptake were classified into personal, interpersonal, group and organizational, societal, and decision-making factors. In terms of vaccination side effects, 35.9% of the participants agreed, 19.8% completely agreed, and 29.5% agreed that these side effects were seen in the long term. Also, 26.5% and 24.8% of adults reported that they agreed and totally agreed, respectively, that natural immunity was better than vaccine immunity. Consistent with our finding, the study of Merkelbach et al. implied that fear of side effects (38.1%) and fear of needles (23.6%) were the most common and strongest barriers in terms of COVID-19 vaccination (36). It is better to provide adults with additional information through the media about vaccination effects on mortality rates to decrease barriers to COVID-19 vaccine uptake. In a study, a high prevalence of vaccine hesitancy was associated with fear of the serious consequences of vaccination and its side effects (31).

In terms of interpersonal factors, 25.2% of participants had no idea, and 15.1% stated that they knew people who had been vaccinated twice yet became ill with COVID-19 disease and died. A lack of long-term studies on the barriers to COVID-19 vaccination (group and organizational variables) was reported by 27.5% of participants. When considering societal and decision-making considerations, 20.1% of people agreed with the statement, “It is advertised in cyberspace that the vaccine is harmful.” The classification of COVID-19 vaccine obstacles reveals that to overcome them, all these elements must be considered, not just individual opinions. The low rate of complete COVID-19 vaccination could be a warning sign that other factors are being overlooked. Additionally, policymakers and affiliated organizations can facilitate COVID-19 vaccination.

Barriers to COVID-19 vaccination are also associated with occupation, income, and marital status. These findings are consistent with the other studies (37, 38). A study showed that socioeconomic status was a determinant of actual COVID-19 booster vaccine acceptance (32). Therefore, in order to increase the COVID-19 vaccination rate, it is necessary to focus on socioeconomic factors and individuals with low socioeconomic status.

In this study, those who had previously received the COVID-19 immunization had higher HL and ML scores. Those who had previously gotten the COVID-19 vaccine also encountered fewer obstacles. This is likely because those who have suffered from deadly infectious diseases are more likely to be motivated to adopt preventive behaviors, such as getting the vaccine. Such individuals can contribute valuable insight to help make health programs even better.

Importantly, HL was positively associated with ML. Barriers to the COVID-19 vaccine were significantly correlated with HL and ML. Previous studies demonstrated that health beliefs, social media influence, HL, and trusting invalid information sources were predictors for planned and actual booster COVID-19 vaccine acceptance (11, 32, 39). Wong et al. (30) showed that positive attitudes toward social media were associated with lower levels of vaccine hesitation in regards to the COVID-19 vaccine booster. Therefore, improved HL and ML can aid in making sense of social media content and boosting the uptake of the COVID-19 vaccination. A study's findings suggested that misinformation and vaccine safety perceptions influenced people's willingness to get the COVID-19 vaccine (29), highlighting the need to trust the information provided by the media. These findings contribute new evidence of the relationship between HL and ML and barriers to COVID-19 vaccine uptake, which could be a guideline for enhancing HL and ML as a necessity for COVID-19 vaccine acceptance and COVID-19 disease control. Importantly, in order to control epidemics and pandemics, in the information and technology era, the role of HL and ML should not be ignored.

The result of our study indicated that demographic characteristics accounted for 8.2% of the variation in barriers to the COVID-19 vaccine; that is, the demographic variables explain 8.6% of the variation in barriers to the COVID-19 vaccine. Monthly income and marital status were statistically associated with low barriers. HL as predictor variables explained an additional 14.4% of the variation in barriers to the COVID-19 vaccine, and ML explained an extra 9.2%. In total, demographic characteristics, HL dimensions, and ML were able to explain 31.8% of the variation in barriers to the COVID-19 vaccine. These results are consistent with those of a study conducted in the United States that found a correlation between vaccine reluctance and factors such as gender, race (particularly African-American and American Indian/Alaska Native), smoking, socioeconomic status, regular use of social media, food insecurity, and lack of access to healthcare (40). Researchers found that people's knowledge of the COVID-19 vaccine was linked to their vaccination history, age, level of education, and even, maybe, gender (41). According to another American study, HL serves as a source of wealth when dealing with a pandemic since it allows for the speedy acquisition of accurate knowledge, the comprehension of social hazards, and the adoption of restrictive government laws and protective behaviors (42). It appears that concentrating on the demographic characteristics of adults, enhancing HL and ML, and developing media-based programs can assist in overcoming barriers and gaining control of COVID-19. The success of health and disease prevention programs depends on considering all the factors identified and effective in the spread of diseases and health problems.

### 4.1. Study limitations

There were some limitations in this research. This research was a cross-sectional study. Hence, it cannot show causal relationships between variables. The barriers to COVID-19 vaccine uptake were investigated in Iran and it may be limited generalizability to other countries. In addition to, these barriers it may not be all the barriers to COVID-19 vaccine uptake. Therefore, it is suggested to be conducted the more studies in the future.

### 4.2. Implications for future research and strengths

One of the strengths of this study is the classification of various barriers to receiving the COVID-19 vaccine in adults, which can be a guideline for policymakers and healthcare providers to design targeted programs. To overcome the barriers to COVID-19 vaccine uptake, it is necessary to address all these factors, not just individual factors and beliefs. In addition, the role and relationship between HL and ML, which were determinants of vaccine uptake barriers, have been assessed. Notably, the results of this study can assist government policies in responding to crises induced by infectious diseases and pandemics.

## 5. Conclusion

The results of the present study revealed that the full COVID-19 vaccination rate was low among Iranian adults. This can be a critical factor hindering efforts to control the COVID-19 pandemic in Iran. In addition, the findings indicate that diverse strategies are required to increase COVID-19 vaccination rates due to the multifactorial nature of the barriers to vaccination, which include personal, interpersonal, group, organizational, societal, and policy-making factors. These findings are required for health policymakers and healthcare providers to design community-based interventions to reduce barriers to adult COVID-19 vaccination. There was a significant correlation between COVID-19 vaccine barriers and the prevalence of HL and ML. Enhancing vaccine HL and ML education for adults and increasing vaccine confidence are crucial.

## Data availability statement

The raw data supporting the conclusions of this article will be made available by the authors, without undue reservation.

## Ethics statement

The studies involving humans were approved by Sarab Faculty of Medical Sciences (IR.SARAB.REC.1401.003). The studies were conducted in accordance with the local legislation and institutional requirements. The participants provided their written informed consent to participate in this study.

## Author contributions

SR and TB conceptualized and designed the study and drafted the initial manuscript. SP carried out the statistical analysis. KMC drafted the initial manuscript and reviewed the manuscript. All authors approved the final manuscript as submitted and agreed to be accountable for all aspects of the work.
